# Effects of deoxynivalenol (DON) and its microbial biotransformation product deepoxy-deoxynivalenol (DOM-1) on a trout, pig, mouse, and human cell line

**DOI:** 10.1007/s12550-017-0289-7

**Published:** 2017-07-24

**Authors:** Elisabeth Mayer, Barbara Novak, Alexandra Springler, Heidi E. Schwartz-Zimmermann, Veronika Nagl, Nicole Reisinger, Sabine Hessenberger, Gerd Schatzmayr

**Affiliations:** 1 BIOMIN Research Center, Technopark 1, 3430 Tulln an der Donau, Austria; 2 Christian Doppler Laboratory for Mycotoxin Metabolism, Center for Analytical Chemistry, Department of Agrobiotechnology (IFA-Tulln), University of Natural Resources and Life Sciences, Vienna, 3430 Tulln an der Donau, Austria

**Keywords:** Cell culture, Cytotoxicity, Contamination, Feedstuff, Metabolite, Transformation, Toxicity, Cell lines, Fish, In vitro

## Abstract

Deoxynivalenol (DON), a trichothecene produced by various *Fusarium* species, is one of the most prevalent food- and feed-associated mycotoxins. The effects of DON and deepoxy-deoxynivalenol (DOM-1) were assessed in five different cell lines from different tissues and species starting from the first line of defense, the trout gill (RTgill-W1) and pig intestinal cells (IPEC-1 and IPEC-J2) over immune cells, as second line of defense (mouse macrophages RAW 264.7) to human liver cells (HepG2). Viability was assessed with a WST-1 assay, except for RTgill-W1, where a neutral red (NR) and sulforhodamine B (SRB) assay was performed. Additionally, more sensitive parameters, such as interleukin-, nitric oxide (NO)-, and albumin-release were determined. Viability was affected by DON at concentrations starting at 10 μmol/L (RTgill-W1), 0.9 μmol/L (IPEC-1), 3.5 μmol/L (IPEC-J2), and 0.9 μmol/L (HepG2), whereas DOM-1 did not have such an effect. Additionally, NO was decreased (0.84 μmol/L DON), whereas interleukin (IL)-6 was increased (0.42 μmol/L DON) in lipopolysaccharide (LPS)-stimulated DON-, but not DOM-1-treated RAW cells. Tumor necrosis factor (TNF)-α release, however, was not affected. Interestingly, albumin secretion of HepG2 cells was decreased by both DON and DOM-1 but at a much higher concentration for DOM-1 (228 versus 0.9 μmol/L for DON). 98.9% of DOM-1 was retrieved by liquid chromatography tandem mass spectrometry at the end of the experiment, proving its stability. In this study, IL-6 was the most sensitive parameter, followed by NO and albumin release and viability for HepG2 and IPEC-1.

## Introduction

Mycotoxins, toxic secondary metabolites of fungi, are a severe problem in agriculture and animal husbandry. Worldwide surveys revealed their widespread prevalence, with up to 72% of agricultural commodities being contaminated (Schatzmayr and Streit 2013). Deoxynivalenol (DON), a type B trichothecene produced by various *Fusarium* spp., is less toxic than some of its related trichothecenes (e.g., nivalenol, T-2 toxin), but still the most prevalent and economically most important mycotoxin in cereal production. Maximum levels and/or guidance values regulating its concentrations in food and feed have therefore been established (European Commission 2006).

DON can be biotransformed by different anaerobic ruminal or intestinal microbes (McCormick 2013). One example for a microbial biotransformation product is deepoxy-deoxynivalenol (DOM-1), which was first described in rats and mice by Yoshizawa et al. (1983) and is formed through cleavage of the 12,13-epoxy ring by bovine rumen microorganisms, such as Genus *novus* (formerly *Eubacterium*) species *novus* BBSH 797 of the *Coriobacteriaceae* family (Fuchs et al. 2002). BBSH 797 is the first-ever microorganism to be cultured, produced, and authorized for its use as a feed additive (European Commission 2013; EFSA 2013a). With the use of these feed additives, DOM-1 gains importance and food safety has to be assured (European Commission 2013). Few studies on DOM-1 are available and regulatory limits for DON metabolites, such as DON glucuronides or DON sulfonates, have not yet been set due to lack in data for absorption and toxicity (EFSA 2013b). For the parent toxin DON, the situation is different, as it has been studied for decades. In general, DON leads to a decrease in feed intake, reduced weight gain, and higher susceptibility to bacterial infections in animals (CAST 2013). Its toxicity on terrestrial animals, especially poultry and pigs, is well documented (Broekaert et al. 2016; Schwartz-Zimmermann et al. 2015). Effects on aquatic animals are however poorly studied, focusing on in vivo studies, assessing only growth and weight (Anater et al. 2016). Due to expansion of the aquaculture industry and the rising costs of fish meal, the use of plant-derived proteins—such as soy bean and other grains as alternative protein sources—quickly increased their demand (Fry et al. 2016). Accordingly, the risk of introducing mycotoxins into animal feed has increased as well, resulting in elevated costs for fish production and decreased animal health. Most investigations have focused on aflatoxin B1 due to its particularly high toxicity (Dirican 2015). The potential effect of DON, despite its frequent occurrence in aquaculture feeds (Gonçalves et al. 2016), has gained increased interest in the last years (Tolosa et al. 2014, Greco et al. 2015, Pietsch et al. 2015, Pelyhe et al. 2016). The European Commission sets the maximum DON concentration at 5 mg/kg for fish feed, which is over 5.5 times greater than the maximum suggested concentration for pig feed (0.9 mg/kg) (European Commission 2006). High DON sensitivity has already been observed in rainbow trout, where DON significantly decreased weight gain, feed intake, and feed efficiency at concentrations above 0.5 mg/kg DON in feed (Hooft et al. 2011). Information about the in vitro effects of DON on fish cells is scarce (Hooft et al. 2011) and effects of DOM-1 have never been assessed in a fish cell line. As the actual concentrations encountered by fish stocks due to agricultural run-off in lakes and rivers are unknown (Hoerger et al. 2009) and water-soluble mycotoxins, like DON, can accumulate in aquaculture, additional research on the effects of DON and DOM-1 is required to facilitate good husbandry practice and to ensure animal welfare.

In contrast to fish, the effects of DON on swine- and pig-derived cells have been studied extensively (Dänicke et al. 2010; Wan et al. 2013). DON compromises gut barrier function, reduces expression of tight junction proteins (Pinton et al. 2012; Springler et al. 2016b), and downregulates multiple transporter systems in enterocytes, impairing nutrient absorption (Ghareeb et al. 2015; Maresca 2013). It is quickly absorbed in the upper part of the porcine gastrointestinal tract (Dänicke et al. 2004b; Grenier and Applegate 2013) and is only moderately biotransformed to DOM-1 by intestinal microbiota in the hindgut (Nagl et al. 2014). Therefore, the effects of DON and DOM-1 were studied and compared in proliferating intestinal porcine epithelial cell lines, IPEC-1 and IPEC-J2.

Immune cells, such as macrophages, T cells, and B cells, present important targets for DON. On a cellular level, inhibition of protein synthesis is regarded as the main effect (Ehrlich and Daigle 1987). Quickly proliferating cells, such as immune cells, with a high protein turnover, are therefore especially sensitive to this mycotoxin. Depending on dose, exposure, frequency, and timing, DON can either stimulate or suppress immunological parameters, such as immunoglobulins and cytokines (Pestka et al. 2004). Macrophages play a major role in host defense against infections through production of mediators such NO, hydrogen peroxide, and cytokines (Lorsbach et al. 1993). Therefore, using murine macrophages, we assessed the effects of DON on viability and NO release and compared these to the release of the cytokines IL-6 and TNF-α.

Following intestinal absorption, DON reaches the liver via the portal vein. As both, intestine and liver with their quickly proliferating cells, have a high protein turnover rate (Savard et al. 2015), they are considered as DON-sensitive organs (Ueno 1984). Following oral consumption of DON, negative effects on liver and serum parameters were found (D’Mello et al. 1999). In the porcine liver, DON concentrations reached maximal values of 4.8 ng/g (~0.1 μmol/L), irrespective of the inclusion rate in the feed (0.55–1.23 mg/kg). DOM-1, however, was only detected in the liver (0–2.4 ng/g (~0.0033 μmol/L) (Döll et al. 2008). To assess the effect of DON on liver cells and due to a lack of a commercially available hepatocellular cell line for pigs, a human hepatocellular cell line (HepG2) was used to compare the effects of DON and DOM-1 on viability and albumin release. Albumin is synthesized in the liver and comprises about one-half of the blood serum protein.

The aim of the study was to assess the effects of DON and DOM-1 on five different cell lines of different animal origin, starting from the first line of defense, the gill and intestinal cells over immune cells, as second line of defense, to liver cells. Particularly, studies of DOM-1 cytotoxicity are scarce and its stability in a culture system as well as its effects on a fish cell line has never been assessed before. Viability was determined with a WST-1 (water-soluble tetrazolium) assay, except for RTgill-W1, where a NR and SRB assay had to be performed. Additionally, more sensitive parameters, such as interleukin- (IL-6 and TNF-α), NO-, and albumin-release, were assessed.

## Materials and methods

### Chemicals and reagents

HyClone Leibovitz L-15 was purchased from GE Healthcare Life Sciences, Marlborough, MA, USA, DMEM/Ham’s F12 and DMEM (4.5 g/L glucose) were purchased from Biochrom AG, Berlin, Germany, and RPMI, penicillin-streptomycin (P/S), and HEPES (4-(2-hydroxyethyl)-1-piperazineethanesulfonic acid) were purchased from Sigma-Aldrich, St. Louis, MO, USA. L-glutamine (L-glut), insulin-transferrin-selenium (ITS), epidermal growth factor (EGF), and fetal bovine serum (FBS) were purchased from Gibco™, Life Technologies, Carlsbad, CA, USA.

DON (Biopure, Romer Labs®, Tulln, Austria) was dissolved in distilled water (6.75 mmol/L) and further diluted with the respective media. DOM-1 (Biopure, Romer Labs®, Tulln, Austria) was obtained in acetonitrile (180.1 μmol/L), evaporated with nitrogen, and further diluted with the respective media. Contaminations of 0.1–0.2% DON and 3% iso-DOM-1 were detected via LC-MS/MS. The calculated solubility (Marvin Software Version 17.9.0, 2017, ChemAxon, (http://www.chemaxon.com)) in water at pH 7 was 14.51 mg/mL (~49 mmol/L) for DON and 11.15 mg/mL (~40 mmol/L) for DOM-1, thus, used concentrations were far below the solubility threshold.

### Cell culture

RAW 264.7 (ATCC® TIB71™) and RTgill-W1 cells (ATCC® CRL. 2523™) were obtained from ATCC (American Type Culture Collection, Manassas, VA, USA). IPEC-1 (ACC-705), IPEC-J2 (ACC-701), and HepG2 (ACC-180) were purchased from DSMZ (German Collection of Microorganisms and Cell Cultures, Braunschweig, Germany). The cells were cultured in respective cultivation media recommended by the supplier and maintained in monolayers in 75-cm^2^ culture flasks (Starlab, Hamburg, Germany) in a humidified incubator at designated temperatures (Table 1). Viability was assessed in triplicates in 96-well plates (Eppendorf, Hamburg, Germany), if not stated otherwise. Mycoplasma tests were performed bimonthly to confirm that cells were free of mycoplasma contamination (Venor® GeM Mycoplasma Detection Kit; Minerva Biolabs, Berlin, Germany).

**Table 1:** Cell lines with the respective medium and DON and DOM-1 concentrations

Cell line	Distributor acc. no.	Temp.	Cultivation medium	Seeding density (cells/well)	DON (μmol/L)	DOM-1 (μmol/L)	Treatment	Assay
RTgill-W1	ATCC® CRL. 2523™	19 °C	HyClone Leibovitz L-15 + 2.05 mmol/L L-glut + P/S + 10% FBS	3.0 × 10^5^	0.125–40	0.125–40	48 h	NR/SRB
IPEC-1	DSMZ ACC-705	39 °C	DMEM/Ham’s F12 (1:1) + 1% ITS +5 ng/mL EGF + 2.5 mmol/L L-glut + 16 mmol/L HEPES + 10% FBS	1.5 × 10^4^	0.2–6.9	0.2–228	24 h	WST-1
IPEC-J2	DSMZ ACC-701			1.2 × 10^4^				
RAW 264.7	ATCC® TIB71™	37 °C	DMEM (4.5 g/L glucose) + 2 mmol/L L-glut + 10% FBS	5.0 × 10^4^	0.1–0.84	1.78–28.5	24 h	
HepG2	DSMZ ACC-180	37 °C	RPMI + 10% FBS	3.5 × 10^4^	0.2–3.5	0.2–228	24 h	

*acc. no.* accession number, *Temp* cultivation temperature, *L-glut* L-glutamine, *P/S* penicillin/streptomycin, *FBS* fetal bovine serum, *ITS* insulin-transferrin-selenium, *EGF* epidermal growth factor, *HEPES* 4-(2-hydroxyethyl)-1-piperazineethanesulfonic acid, *NR* neutral red, *SRB* *sulforhodamine B*, *WST-1* water-soluble tetrazolium salt-1

#### RTgill-W1

The rainbow trout (*Oncorhynchus mykiss*) epithelial gill cell line RTgill-W1 was cultured according to Table 1 in normal atmosphere (ambient gas composition 21% O_2_, 78% N_2_, and 0.04% CO_2_) (Bols et al. 1994). The cells were seeded and cultured in 96-well flat-bottom plates for 72 h and subsequently treated with DON or DOM-1 for 48 h. A NR and SRB assay (both Aniara, West Chester, OH, USA) were performed, as the WST-1 assay (which was applied for all other cell lines) as well as the 24-h incubation time did not fulfill the manufacturer’s performance standards (OD of 1 for cell control was not reached). Experiments were performed in three (NR) and four (SRB) independent experiments.

### NR and SRB assay

A dual-parameter assay, using a NR assay, targeting the lysosomal activity, followed by a SRB assay, targeting the total protein content (and not the de novo protein synthesis), was carried out to assess cell viability. Both assays were performed according to the manufacturer’s instructions. Briefly, NR was added (1:100 in medium), incubated for 3 h, fixed for 1 min, and subsequently dissolved. Absorbance was measured at 540 nm, with a reference filter of 690 nm. Thereafter, a SRB assay was performed in the same well. In short, cells were washed, fixed and incubated with SRB for 15 min, washed again, and dissolved, and absorbance was measured at 540 nm, with a reference filter of 690 nm.

#### IPEC-1 and IPEC-J2

To compare the spontaneously immortalized, non-transformed, intestinal porcine epithelial cells IPEC-1 and IPEC-J2 (Gonzalez-Vallina et al. 1996; Schierack et al. 2006), they were cultured according to Table 1 and cultivated in vitro for a maximum of 15 passages. Cells were seeded in 96-well flat-bottom plates, incubated for 48 h, and then treated with DON or DOM-1 for 24 h. Viability was assessed via the WST-1 assay and expressed as percent compared to a joint cell-control of three independent experiments, which was set to 100.

### WST-1 assay

Cell proliferation reagent WST-1 (4-[3-(4-iodophenyl)-2-(4-nitrophenyl)-2H-5-tetrazolio]-1,3-benzene disulfonate) assay (Roche, Switzerland) was performed according to the manufacturer’s instructions. Briefly, the cells were incubated with a 5% WST-1 solution in medium for a maximum of 4 h at designated temperatures and quantified via spectrometry at 450 nm. The development of formazan dye correlates to the number of metabolically active cells in the culture.

#### RAW 264.7

The cells were cultured according to Table 1. RAW 264.7 macrophages were seeded in 96-well flat-bottom plates, incubated for 24 h, and subsequently stimulated with and without LPS from *Escherichia coli* O111:B4 (1 μg/mL) (Sigma-Aldrich, St. Louis, MO, USA) and DON or DOM-1 for a further 24 h. Subsequently, the supernatant was collected for NO and cytokine determination. A WST-1 assay was performed thereafter, to assess viability. Experiments were performed in five independent experiments.

### NO measurement

The amount of NO in supernatants of RAW 264.7 macrophages was determined via the Griess diazotization reaction (Roche, Basel, Switzerland). Fifty microliters of cell supernatant was used for NO determination. The remaining supernatant was stored at −20 °C for cytokine determination. Experiments were performed in five independent experiments.

Absorbance was measured at 540 nm using a microplate reader. Relative NO release was calculated as follows:

1 $$ \mathrm{NO}\kern0.5em \mathrm{release}\left(\%\right)=\left(\left(\mathrm{NO}\kern0.5em \mathrm{concentration}\kern0.5em \mathrm{of}\kern0.5em LPS\hbox{-} \mathrm{treated}\kern0.5em \hbox{-} \kern0.5em \mathrm{NO}\kern0.5em \mathrm{concentration}\kern0.5em \mathrm{sample}\hbox{-} \mathrm{treated}\right)/\mathrm{NO}\kern0.5em \mathrm{concentration}\kern0.5em LPS\hbox{-} \mathrm{treated}\right)\times 100 $$

### Cytokine determination

IL-6 and TNF-α were determined in RAW 264.7 cell supernatant in duplicates by a multiplex bead-based flow cytometric assay (Flow Cytomix™, eBioscience, Austria). Thawed supernatant was centrifuged and 25 μL was treated according to the instructions of the manufacturer and thereafter measured by the flow cytometer Accuri C6™ (BD, Heidelberg, Germany) using the BD Sampler Analysis Software. Standard curves were determined for each cytokine in a range of 27–20,000 pg/mL. Cytokine concentration was expressed as percent compared to a joint LPS-control of three independent experiments, which was set to 100.

#### HepG2

The human hepatocellular carcinoma cell line HepG2 was cultured according to Table 1. For viability assays, HepG2 cells were seeded, incubated for 48 h, and then treated with varying concentrations of DON (0.2–3.5 μmol/L) and DOM-1 (0.2–228 μmol/L) for additional 24 h. A WST-1 assay was performed at the end of the experiment as explained earlier. Experiments were performed in three independent experiments.

### Albumin detection

Albumin concentration in the undiluted supernatant was determined via colorimetric sandwich enzyme-linked immunosorbent assay (ELISA) (Abnova, Taiwan, China). Experiments were performed in duplicate in three independent experiments.

### LC-MS/MS analysis

The RAW 264.7 cells were used as representative cell line to determine DOM-1 concentration after 24 h of incubation. Therefore, RAW cells were treated with RAW medium alone, DOM-1 (1.74 μmol/L) alone, and DOM-1 together with LPS (1 μg/mL) for 24 h, frozen at −80 °C, and then measured by liquid chromatography tandem mass spectrometry (LC-MS/MS). Analyses were carried out on an Agilent 1100 series HPLC system (Waldbronn, Germany). Analytes were separated in gradient elution on an Eclipse XDB-C8 column (150 × 4.6 mm, 5 μmol/L) at 25 °C using methanol/5 mmol/L aqueous ammonium acetate buffer (A 20/80, *v*/*v*; B 95/5, *v*/*v*) as mobile phases. The gradient started with a linear increase from 0 to 100% B within 2 min and continued for 3 min at 100% B. Afterwards, the column was re-equilibrated at 0% B for 2 min, reaching a total run-time of 7 min. The flow rate was 1 mL/min, and the injection volume was 15 μL.

Tandem mass spectrometric analysis was carried out on a 2000 QTrap mass spectrometer equipped with an APCI ion source (SCIEX) in the negative ionization mode. Parameters of selected reaction monitoring transitions (dwell time of 25 ms) were optimized by software-controlled compound optimization and were as follows for DON and DOM-1 that were determined in the form of their acetate adducts: DON quantifier *m/z* 355.1 → *m/z* 59.1 (declustering potential (DP) −16 V, collision energy (CE) −30 eV), DON qualifier *m/z* 355.1 → *m/z* 265.1 (DP −16 V, CE −12 eV); DOM-1 quantifier *m/z* 339.1 → *m/z* 59.1 (DP −21 V, CE −40 eV), DOM-1 qualifier *m/z* 339.1 → *m/z* 249.1 (DP −21 V, CE −16 eV). Ion source settings were as follows: curtain gas 40 psi, source temperature 450 °C, nebulizer gas (GS1) 15 psi, heater gas (GS2) 60 psi, and collisionally activated dissociation (CAD) gas 6. Analyst® software version 1.5.2 was used for instrument control and data analysis.

Analytes were quantified on the basis of neat solvent calibration functions established between 10 (~0.03 μmol/L) and 1000 ng/mL (~3.57 μmol/L) DOM-1. The retrieved DOM-1 was calculated as follows:

2 $$ \mathrm{Retrieved}\kern0.5em DOM\hbox{-} 1\left(\%\right)=\left(DOM\hbox{-} 1\kern0.5em \mathrm{detected}\kern0.5em by\kern0.5em LC\hbox{-} MS/MS/\mathrm{applied}\kern0.5em DOM\hbox{-} 1\left(1.74\ \upmu \mathrm{mol}/\mathrm{L}\right)\times 100\right. $$

### Statistics

Statistical analysis was performed with IBM® SPSS Statistics (Version 22.0, IBM Inc., New York, NY, USA, 2013). Values were analyzed for normality (Shapiro-Wilk) and homogeneity of variance (Levene Statistics). Normally distributed homogenous data were analyzed by analysis of variance (ANOVA) and Dunnett’s *t* test compared to those of the control. Normally distributed but not homogenous data were analyzed by ANOVA and Dunnett’s T3-test. When the assumptions of the ANOVA were not met, a non-parametric Kruskal-Wallis test was used. Significances (*p* < 0.05) were marked with an asterisk.

Viability was calculated by setting the measured OD of the cell control to 100%. When only three independent experiments were present, a joint mean, which was set to 100%, was calculated.

## Results

**Fig. 1:**
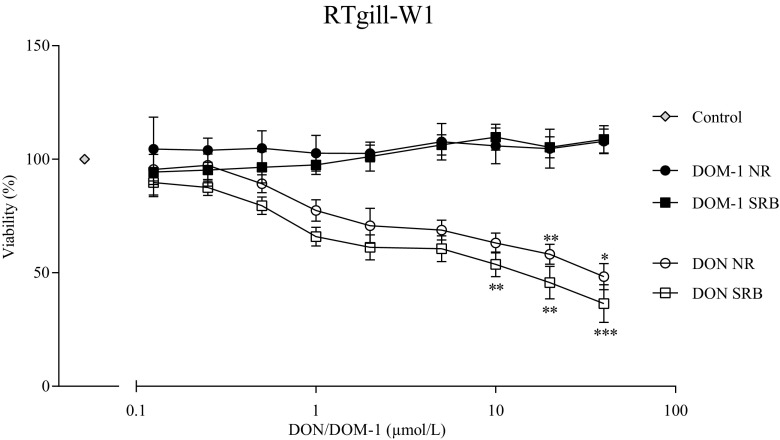
Effect of DON and DOM-1 on viability (%) of the RTgill-W1 fish cell line. RTgill-W1 cells were treated with DON and DOM-1 (0.125–40 μmol/L) for 48 h. A NR (*sphere*) and a SRB (*square*) assay were performed to assess the viability. Data represent mean ± SD, *n* = 3 (NR) and *n* = 4 (SRB). *Asterisks* indicate significant difference from control (*p < 0.05, **p < 0.01, and ***p < 0.001)

The viability of rainbow trout gill cells (RTgill-W1) was determined after treatment with equimolar amounts of DON and DOM-1 (0.125–40 μmol/L) with a SRB and NR assay. A significant effect of DON on cell viability was observed above 10 μmol/L (*p* = 0.008) with the SRB assay and 20 μmol/L (*p* = 0.002) with the NR assay. At the highest tested concentration (40 μmol/L), viability was reduced by 63% ± 7.6 (*p* < 0.001) and 52% ± 5.0 (*p* = 0.018), according to the SRB and NR assay, respectively (Fig. 1).

However, no effect of DOM-1 on cell viability was detected for the range of concentrations tested.

**Fig. 2:**
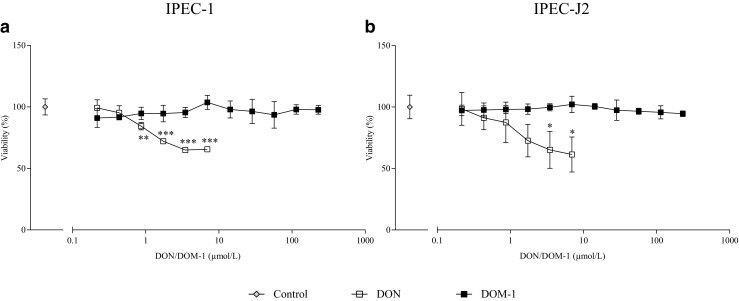
Effect of DON and DOM-1 on viability (%) of the IPEC-1 (*left*) and IPEC-J2 (*right*) cell line. **a** IPEC-1 and **b** IPEC-J2 were treated with DON (0.2–6.9 μmol/L) or DOM-1 (0.2–228 μmol/L) for 24 h. Viability was evaluated with the WST-1 assay and expressed as relative values compared to the control of all independent experiments (set to a joint 100%). Data represent mean ± SD, *n* = 3. *Asterisks* indicate significant differences compared to control (*p < 0.05, **p < 0.01, and ***p < 0.001)

We next assessed the effect of DON (0.2–6.9 μmol/L) and DOM-1 (0.2–228 μmol/L) on the viability of intestinal epithelial cell lines IPEC-1 and IPEC-J2 via the WST-1 assay. DON dose-dependently decreased viability in both cell lines (Fig. 2). At concentrations at and above 0.9 μmol/L DON (IPEC-1) and 3.5 μmol/L DON (IPEC-J2), a significant decrease (*p* < 0.05) in viability was observed. At the highest concentration (6.9 μmol/L DON), viability of IPEC-1 and IPEC-J2 was reduced to 65.6% ± 2.1 and 60.9% ± 8.3, respectively. In contrast, DOM-1, even tested up to 228 μmol/L, did not affect viability (>90%) of either cell line at any tested concentration.

**Fig. 3:**
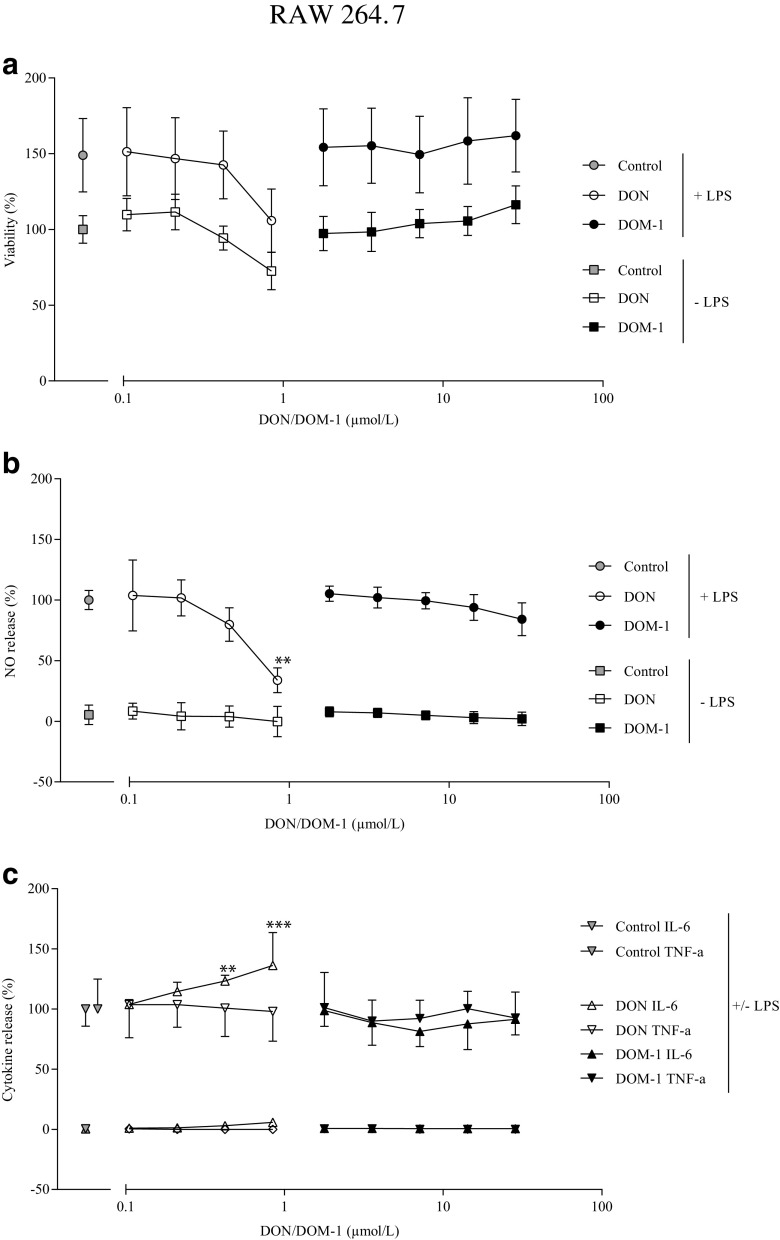
Effect of DON and DOM-1 on **a** viability, **b** NO-, and **c** cytokine release of RAW 264.7 cells. RAW 264.7 cells were treated with DON (0.1–0.84 μmol/L) or DOM-1 (1.78–28.5 μmol/L) with (+) and without (−) LPS (1 μg/mL) (± LPS). After 24 h of incubation with the mycotoxin, a WST-1 assay was performed to assess viability (**a**) and the supernatant was collected for NO determination (**b**) and cytokine (IL-6 (*triangle up*) and TNF-α (*triangle down*) release (**c**). Data represent mean ± SD, *n* = 5 (**a** and **b**), *n* = 3 (**c**). *Asterisks* indicate significant differences from the respective control (**p < 0.01 and ***p < 0.001)

To further assess the effect of DON (0.1–0.84 μmol/L) and DOM-1 (1.78–28.5 μmol/L) on viability, NO, and cytokine release, the murine macrophage cell line RAW 264.7 was used with and without stimulation with LPS (1 μg/mL). LPS was used as a representative molecule to simulate inflammation. Inflammation is an important and vital tool to fight infections. The potential of DON or DOM-1 to suppress inflammatory processes was therefore assessed. Neither DON nor DOM-1 significantly decreased viability at the tested concentrations, regardless of whether LPS was applied or not (Fig. 3a). Compared to untreated (control) cells, viability of LPS-stimulated RAW 264.7 cells was increased by 49% ± 24.0. LPS treatment in the presence of DON or DOM-1 led to a similar ~50% increase of viability.

NO release was induced neither by DON nor by DOM-1 at the tested concentrations in unstimulated cells (Fig. 3b). Values were at the level of the negative control (5.4% ± 8.0) for DON (6.4% ± 4.5) and DOM-1 (4.9% ± 2.4). However, in LPS-stimulated cells (set to 100%), a significant decrease of NO release was observed at the highest DON concentration (0.84 μmol/L) (*p* = 0.001), resulting in a decrease of 66.2% ± 9.6 compared to the LPS-induced NO production (=100%). For DOM-1, no significant reduction even at higher concentrations was observed.

Additionally, supernatants were tested for IL-6 and TNF-α cytokine release (Fig. 3c). DON and DOM-1 alone did not significantly affect cytokine release. Interestingly, a superinduction of the inflammatory cytokine IL-6 was observed in DON-treated cells stimulated with LPS. A significant increase of 23% ± 4.8 (*p* = 0.008) was seen at 0.42 μmol/L and 36% ± 27.4 (*p* < 0.001) at 0.84 μmol/L DON. In contrast, TNF-α levels for DON plus LPS-stimulated cells ranged between 98 and 103%. DOM-1 in the presence of LPS did not influence the cytokine release for IL-6 nor for TNF-α.

**Fig. 4:**
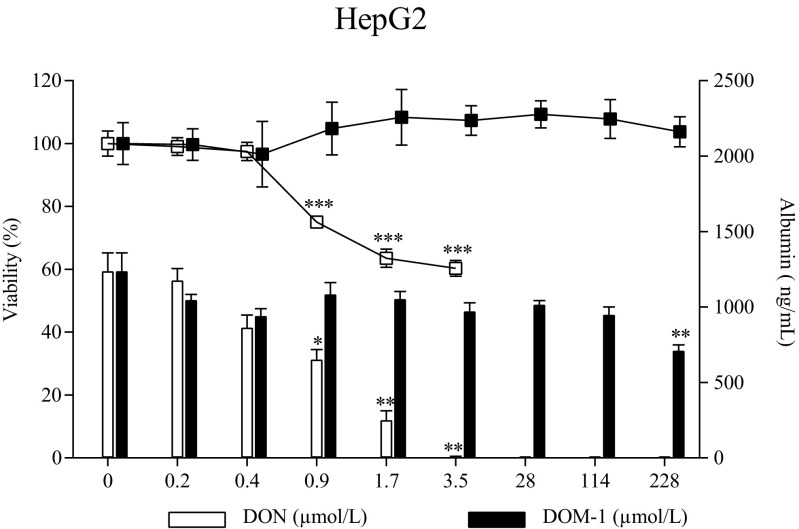
Effect of DON and DOM-1 on viability (%; *left axis*, *squares*) and albumin release (ng/mL; *right axis*, *bars*) of HepG2 cells. HepG2 cells were treated with either DON (0.2–3.5 μmol/L; *white*) or DOM-1 (0.2–228 μmol/L, *black*) for 24 h. **a** Viability was assessed with a WST-1 assay after 24 h of incubation. **b** Albumin production was assessed via ELISA thereof. Data represent mean ± SD, *n* = 3. *Asterisks* indicate significant difference from control (*p < 0.05, **p < 0.01, and ***p < 0.001)

HepG2 cells were stimulated with DON (0.2–3.5 μmol/L) and DOM-1 (0.2–228 μmol/L) for 24 h, followed by a WST-1 assay. DON dose-dependently and significantly decreased viability at increasing concentrations starting at 0.9 μmol/L (*p* < 0.001) (Fig. 4). At the highest tested concentration of 3.5 μmol/L DON, viability was decreased by 39.7% ± 4.0. For DOM-1, no such effect was seen at even higher concentrations.

When albumin production was assessed in the respective supernatant, a similar trend was observed (Fig. 4). Albumin secretion was reduced by 47.3% ± 10.3 (*p* = 0.02) at 0.9 μmol/L DON. At 3.5 μmol/L DON, no more albumin was detected (99.6% ± 0.7 albumin reduction; *p* = 0.015). DOM-1 affected albumin production at the highest concentration (228 μmol/L), where a statistically significant reduction of 42.6% ± 5.9 (*p* = 0.002) was observed.

The stability of DON has been confirmed by many studies (reviewed in (Sobrova et al. 2010)). To assess the stability of DOM-1 in the course of the experiment, RAW 264.7 cells were treated with 1.74 μmol/L DOM-1 in the absence or presence of 1 μg/mL LPS for 24 h. Supernatants were collected and measured by LC-MS/MS (Table 2). For all tested samples, the mean recovery rate was 98.9% ± 2.4 when DOM-1 was present. No DOM-1 was found in the respective medium control (RAW medium).

**Table 2:** Recovery rate of DOM-1 (%) measured by LC-MS/MS after 24 h of incubation. Data represent mean ± SD, *n* = 3

Sample	Retrieved DOM-1			Incubation time
	(μmol/L)		(%)	
DOM-1 (1.74 μmol/L)	1.75 ± 0.07		100.6 ± 4.0	24 h
DOM-1 (1.74 μmol/L) + LPS (1 μg/mL)	1.69 ± 0.07		97.1 ± 4.0	
RAW medium	No peak		0	
Recovery rate (mean ± SD)			98.9 ± 2.4	

## Discussion

The present study portrays the effects of DON and its primary microbial metabolite, DOM-1, on five different cell lines. In that, we are the first to provide the effects of DON and DOM-1 in cells of different species and tissue origin and report various degrees of sensitivity.

To compare effects of DON and DOM-1 after 24 h, viability was assessed with the WST-1 assay, targeting the mitochondrial activity, which, although less sensitive than other assays (Riss et al. 2004), provides a good correlation to in vivo data (Reubel et al. 1987). As the WST-1 assay was not valid for RTgill-W1 cells, most likely due to their low metabolic turnover and proliferation rate (Lee et al. 2009), viability was assessed with a dual-parameter cytotoxicity assay for 48 h, evaluating the lysosomal activity (NR assay) and total protein content (SRB assay). DON affected cell viability at concentrations above 0.9 μmol/L (IPEC-1 and HepG2), 3.5 μmol/L (IPEC-J2), and 10 μmol/L (RTgill-W1). For RAW 264.7 cells, a lowered viability was observed in the presence of DON (~minus 30%, regardless of the LPS stimulation) at the highest concentration (0.84 μmol/L DON), which was, however, not significant. For further experiments, the DON concentration should therefore be increased. DOM-1 did not affect cell viability up to 228 μmol/L. Significant effects on IL-6, NO-, and albumin release were already seen at lower DON concentrations of 0.42 μmol/L (IL-6), 0.84 μmol/L (NO), and 0.9 μmol/L (albumin). The only parameter affected by DOM-1 was albumin which was reduced by ~50% at the highest tested concentration of 228 μmol/L, demonstrating a ~253 times lower susceptibility to DOM-1 than to DON. This negative effect could be ascribed to the 0.1–0.2% DON contamination in the DOM-1 standard, which would already account for 0.23–0.46 μmol/L DON.

Mycotoxin-induced damage to gill cells—which are involved in gas exchange and osmoregulation and constitute the first line of defense in fish against pathogens and toxins—can lead to impairments of the whole organism. According to the recommendation of the European Commission (European Commission 2006), DON levels should not exceed 5 mg/kg in complete feedstuff for fish. In this study, 40 μmol/L DON and equimolar DOM-1 concentrations were tested. RTgill-W1 were least sensitive to DON and viability was differently affected by DON, depending on the observed target. The total protein content (SRB assay) was already reduced at 10 μmol/L DON, whereas lysosomal activity (NR assay) was first affected at 20 μmol/L. The higher sensitivity of the SRB assay has been recently shown by Springler et al. (2016a) and can be explained by the primary toxicological feature of DON, protein synthesis impairment (Springler et al. 2016a). These negative effects of DON could already be seen at 2 μmol/L DON, where a viability reduction of 39% ± 3.6 was observed. Our findings are in accordance with Pietsch et al. (2011), who confirmed reduced viabilities at concentrations above 2.7 μmol/L DON for both applied assays. Living rainbow trout were even more sensitive to DON, as weight gain, feed intake, and feed efficiency were already affected by 0.5 mg/kg DON in feed (Hooft et al. 2011). DOM-1 has never been studied in fish—we are the first to provide insight into the non-cytotoxic effects of DOM-1 on fish cells up to 40 μmol/L.

IPEC-1 and IPEC-J2 are already established models to thoroughly study the intestinal function, especially due to their morphological and functional constitution, as well as their sensitivity to toxins (Nossol et al. 2011). Concentrations up to 6.9 μmol/L DON were used, which would correspond to 2 mg/kg DON in feed according to the calculations of Pinton et al. (2010). A decrease in viability was observed at DON concentrations above 0.9 μmol/L DON for IPEC-1 and 3.5 μmol/L for IPEC-J2, suggesting that IPEC-1 are more sensitive to DON. This is in accordance with Dänicke et al. (2010), where a significant decrease in viability was seen at 0.2 μmol/L DON for IPEC-1 and 0.7 μmol/L DON for IPEC-J2 assessed by MTT assay (Dänicke et al. 2010). In contrast to our study, DON was added directly after cell seeding and for 24 h longer, explaining the higher sensitivity of the cells. In a study of Alassane-Kpembi et al. (2015), reduced viability was seen above 0.5 μmol/L DON with the MTT assay in IPEC-1 (Alassane-Kpembi et al. 2015), which is in accordance to our results. For DOM-1, no decrease in viability was seen, although more than 32 times higher concentrations (up to 228 μmol/L DOM-1) compared to DON were used.

To apply and correlate in vitro to in vivo results, the exposure of the cells has to be taken into account. Intestinal cells in vivo are not permanently subjected to DON, but exposed at time-dependent fluctuations. Orally ingested DON reaches a maximum after 30 min in the plasma, whereas the absorption phase is nearly finished 4 h after DON ingestion (Dänicke et al. 2010; Goyarts and Dänicke 2006). In contrast, the intestinal cell lines are permanently exposed to DON for 24 h, without the protective mucus layer, explaining toxic effects already at lower concentrations compared to in vivo results. A similar decrease in viability was also observed in other cell lines, such as the human intestinal HT-29-D4 cells (Maresca et al. 2002) with a viability decrease at 1 μmol/L DON. In the human adenocarcinoma cell line Caco-2, a decrease of viability was observed at 0.7 μmol/L (Sergent et al. 2006), 3.4 μmol/L (Cetin and Bullerman 2005), and 1.38 μmol/L (He et al. 2015), which is in accordance to our results.

In serum, DON can affect blood cells such as macrophages. A comparable and linear relationship between DON consumption and retrieval in blood has been reported by Döll et al. (2003) and Dänicke et al. (2004a, b). DON concentrations up to 100 ng/mL (~0.3 μmol/L) (Coppock et al. 1985) and 325 ng/mL (~1 μmol/L) (Prelusky et al. 1988) were detected. The macrophage cell line RAW 264.7 was chosen for assessing effects of DON on blood cells. In this study, DON either enhanced (IL-6 increase) or suppressed (decrease in NO release) critical macrophage functions. While mediators, such as NO, play a key role in immunity, vasodilation, inflammation, thrombosis, and neurotransmission (Fukuo et al. 1995), it also has been shown that activated macrophages suppress the NO release and simultaneously increase the release of the pro-inflammatory cytokines (e.g., TNF-α and IL-6) (Ji et al. 1998). According to Shi et al. (2009), low and moderate DON exposure induced pro-inflammatory gene expression, but repeated exposures to high concentrations induced cell death (Shi et al. 2009). In this study, the viability of DON-treated RAW 264.7 cells was not significantly decreased at the tested concentrations, even though a reduction of 27% for unstimulated and 44% for stimulated cells was seen. Vandenbroucke et al. (2009) saw significant reduction in viability (−15%) already at 0.84 μmol/L DON in porcine macrophages (Vandenbroucke et al. 2009). For DOM-1, no viability reduction was observed in our study, neither with nor without LPS stimulation, even though ~34 times higher concentrations were used. Protein synthesis inhibitors, like anisomycin and cycloheximide, or DON, have been shown to superinduce cytokine gene expression and secretion (Ghareeb et al. 2015). The phenomenon of superinduction is not fully understood yet but can partially be explained by a decreased messenger RNA (mRNA) degradation through inhibition of translational repressor proteins, a decreased synthesis of labile selective nucleases (Azcona-Olivera et al. 1995), or a direct stimulation of intracellular signaling pathways (Mahadevan and Edwards 1991). In RAW 264.7 cells, TNF-a and IL-6 superinduction has been observed after DON exposure (Ji et al. 1998, Zhou et al. 1999). Wong et al. (1998) however, only detected IL-6 but not TNF-a superinduction (Wong et al. 1998). This could be explained by the increased half-life for IL-6 mRNA (60 min) compared to TNF-a mRNA (25 min)—which was even further increased in the presence of DON (~0.84 μmol/L) (Wong et al. 2001), as well as the increased stability of IL-6 (24 h) compared to TNF-a (6 h) (Sugita-Konishi and Pestka 2001).

When cytotoxicity of DON was assessed in HepG2 cells, a decrease in viability was already observed at 0.9 μmol/L. These liver cells seem to be as sensitive as the IPEC-1 cell line in these experiments. Other studies that assessed the effect on DON on HepG2 cells found varying IC50 values of 1.9 μmol/L (Nielsen et al. 2009a), 1.89 μmol/L (Nielsen et al. 2009b), and 28.2 μmol/L after 48 h of incubation (Cetin and Bullerman 2005). Nielsen et al. (2009b) observed a viability reduction of 25% at 1 μmol/L DON which is in accordance to our observation (24.9% ± 1 reduction at 0.9 μmol/L DON). When albumin release was assessed in the respective supernatant, a similar picture compared to the toxic effect of DON was observed. A significant decrease was observed at 0.9 μmol/L. Interestingly, albumin was the only parameter that was affected by DOM-1. However, the concentration of 228 μmol/L DOM-1 was comparatively high and would correspond to 68 mg/kg DON in the feed, according to the calculation of Pinton et al. (2010). The effect cannot be explained by DON contamination of the DOM-1 standard, which was evaluated by LC-MS/MS.

Animals with a high bacterial load at the upper part of the gastrointestinal tract (such as ruminants with bacteria in rumen and birds with bacteria in the crop) are able to bacterially biotransform DON into less toxic metabolites, such as DOM-1, leaving the animal less affected by the toxin. In monogastric animals (e.g., humans/pigs/rodents) however, ingested DON can directly enter the blood crossing the intestinal epithelium. In these animals, detoxification strategies, such as intestinal biotransformation, were predominately observed in the lower gastrointestinal tract, hence only marginal detoxification is possible by bacterial transformation (Maresca 2013). With the use of feed additives that contain DON- to DOM-1-transforming bovine rumen bacteria, such as Genus *novus* (formerly *Eubacterium*) species *novus* BBSH 797 of the *Coriobacteriaceae* family, DOM-1 gains relevance in feed again and food safety has to be assured (European Commission 2013). The mode of action of the de-epoxidation of DON was proven in in vitro and in vivo experiments (Ghareeb et al. 2015; Schatzmayr et al. 2006).

For DOM-1, studies on the cytotoxic effect are scarce. Kollarczik et al. (1994) were the first to show that the metabolite, biotransformed in the bowel after anaerobic incubation with DON, significantly decreased the cytotoxicity of DON in pig kidney cells. Although some studies regarding the effects of DOM-1 are available (Dänicke et al. 2011; Mishra et al. 2014; Nasri et al. 2006; Sundstol Eriksen et al. 2004), a thorough comparison of five different cell lines has never been conducted. Additionally, the stability of DOM-1 via LC-MS/MS has never been assessed before.

Although susceptibility to DON varies between animal species, which can be explained by differences in absorption, metabolism, and elimination of DON (Pestka 2010; Pestka and Smolinski 2005) as well as in differences in the location of bacteria, susceptibility can also vary greatly among cell types. A direct transfer of results from in vitro to in vivo is therefore always challenging.

This study provides, for the first time, effects of DON and DOM-1 on five different cell lines of different animal origin, starting from the first line of defense, the gill and intestinal cells over immune cells to liver cells. Overall, DON reduced viability in RTgill-W1 (10 μmol/L), IPEC-1 (above 0.9 μmol/L), IPEC-J2 (above 3.5 μmol/L), and HepG2 cells (above 0.9 μmol/L), whereas DOM-1 did not have such an effect. The cell parameter that was affected by the lowest DON concentration of 0.42 μmol/L DON in LPS-stimulated RAW 264.7 cells was IL-6, followed by the NO release at 0.84 μmol/L in LPS-stimulated RAW 264.7 cells. Albumin secretion of HepG2 cells was the only parameter decreased by both, DON and DOM-1, but at a much higher concentration for DOM-1 (228 versus 0.9 μmol/L for DON).

## Abbreviations

DOM-1: deepoxy-deoxynivalenol
DON: deoxynivalenol
IPEC: intestinal porcine epithelial cells
LC-MS/MS: liquid chromatography tandem mass spectrometry
LPS: lipopolysaccharide
NO: nitric oxide
NR: neutral red
SRB: sulforhodamine B
TNF: tumor necrosis factor
WST-1: water-soluble tetrazolium salt-1
